# Daratumumab Treatment for “Truly Frail” Elderly Myeloma Patients

**DOI:** 10.3390/life14030389

**Published:** 2024-03-15

**Authors:** Yuichi Horigome, Kazuhito Suzuki, Takahiro Suzuki

**Affiliations:** 1 Department of Hematology, Kitasato University Graduate School of Medical Sciences, Sagamihara 252-0373, Kanagawa, Japan; tasuzuki@med.kitasato-u.ac.jp; 2 Division of Clinical Oncology and Hematology, Department of Internal Medicine, The Jikei University School of Medicine, Minato City 105-8461, Tokyo, Japan, Japan; kaz-suzuki@jikei.ac.jp; 3 Department of Hematology, Kitasato University School of Medicine, Sagamihara 252-0374, Kanagawa, Japan

**Keywords:** multiple myeloma, daratumumab, frail, elderly, quality of life

## Abstract

Remarkable advancements have been made in the treatment outcomes of multiple myeloma (MM) patients; however, for frail elderly patients, these treatment outcomes are still insufficient. Elderly MM patients are increasing, as are their treatment regimens. There is a heightened demand to assess these patients in order to provide optimized treatments. While continuous treatment is more common for MM patients when compared to fixed-duration treatment, due to the risk of treatment interruption causing reduced survival rates, effectiveness and safety are essential. Treatment goals vary for each patient, but must preserve their quality of life (QOL). When planning treatments for these patients, frailty evaluation is increasingly emphasized as a stratification factor which helps develop accurate screening tools. Daratumumab (DARA) therapy, used globally, is not only effective in frail elderly MM patients, but also has QOL benefits. Proficiency in utilizing DARA regimens is potentially advantageous for patients not included in clinical trials, and innovative usage can further broaden its scope. The development of tools to accurately assess frailty and the establishment of optimal treatments for frail elderly MM patients are imperative. This review is an overview, challenging the frailty assessments for MM patients, re-examining the evidence for DARA regimens in frail elderly MM patients, and discussing potential areas for improvement.

## 1. Introduction

**Figure 1:**
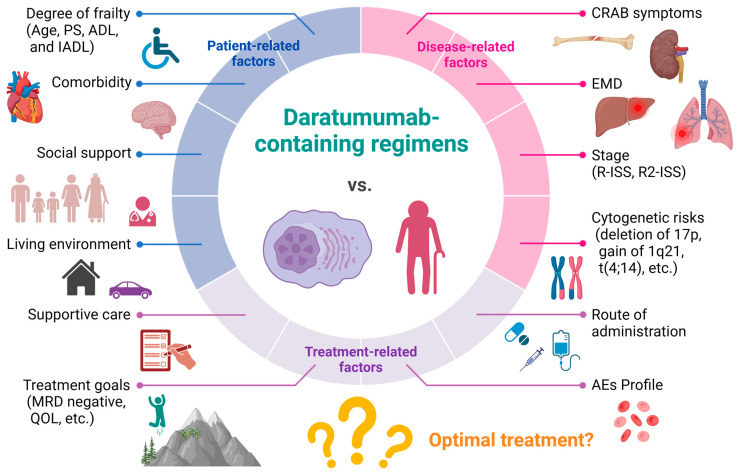
Three factors to consider when determining the treatment approach for frail elderly MM patients. Accurately assessing the degree of frailty is necessary to determine the treatment approach for frail elderly MM patients. As shown, in addition to patient-related factors, such as comorbidities, caregivers, and family support, various factors including disease- and treatment-related factors must be thoroughly evaluated. Efforts must be made to provide the most suitable treatment for each patient. CRAB: hypercalcemia, renal failure, anemia, bone disease. Created with BioRender.com (accessed on 29 January 2024).

The development of new drugs has dramatically improved the treatment outcomes for multiple myeloma (MM), but the improvement in transplant-ineligible MM (TIE-MM) patients is limited [1]. In recent years, due to global aging, it is expected that the number of elderly MM patients will further increase in the future [2]. In other words, it is anticipated that clinicians will be strongly urged to adequately evaluate a wide variety of elderly (≥70 years) MM patients, offering them the most suitable treatment, while avoiding any inadequate and excessive treatment. It is necessary to comprehensively consider all factors, including disease-related factors, such as disease stage, the presence of high-risk chromosomal abnormalities, the presence of extramedullary disease, and the presence of amyloidosis, as well as patient-related factors, such as performance status (PS), frailty, activities of daily living (ADL), comorbidities, the patient’s living environment, caregivers, the patient’s place of residence, means of transportation for clinic visits, and treatment-related factors, such as drug selection considering comorbid conditions, the selection of treatment regimens based on understanding of typical adverse events (AEs), and any drugs used previously in prior lines of therapies [3]. Furthermore, for the introduced treatment regimen to be effective for frail elderly MM patients, it is important not only to enhance treatment intensity in pursuit of effectiveness, but also to be conscious of maintaining and, whenever possible, improving their QOL. Ultimately, therefore, achieving a balance between both aspects is crucial (Figure 1).

Elderly patients with TIE-MM, especially those who are frail, face significant challenges as they undergo successive lines of therapy. It has previously been reported that, with each additional treatment, outcomes tend to worsen, and attrition rates can reach up to 50% per line of therapy [4]. Therefore, it is suggested that for elderly MM patients, it is important to introduce highly effective treatment regimens early, as demonstrated in trials such as the MAIA trial [5,6] and POLLUX trial [7,8,9,10]. However, treatment interruptions due to AEs during initial therapy have also been demonstrated to lead to decreased survival rates in elderly MM patients [11]. Therefore, it is important to have a thorough understanding of the potential AEs that could occur with each treatment regimen, manage each effectively.

Due to the heterogeneous nature of the elderly population, establishing high-quality evidence for them is challenging. Moreover, in clinical trials, patients with poor health conditions are generally excluded, i.e., when planning treatment for elderly MM patients, providing treatment based on sufficient evidence is difficult. As a result, the current situation involves a trial-and-error approach for each individual case.

To address this challenge, many clinical trials are exploring the assessment of frailty and its stratification as a means to overcome this limitation. The frailty index is considered a powerful indicator for predicting the clinical course of individual elderly MM patients, and it is widely used in many clinical trials with analyses ongoing. If treatment schedules, drug dosages, adjustments to supportive therapies, treatment durations, and the timing for transitioning to subsequent treatments could be determined based on the frailty score, it has the potential to be extremely beneficial in the context of practical clinical scenarios. However, it should be noted that there is presently a shortage of data for the accurate treatment decisions based on frailty, and the development of screening tools to properly assess frailty is still terribly inadequate.

In this review, we discuss the overview and challenges of frailty assessment tools for MM patients, examine the evidence for daratumumab (DARA)-containing regimens in elderly patients with newly diagnosed MM (NDMM) or relapsed/refractory MM (RRMM), and further explore strategies to maximize the benefits of DARA-containing regimens for frail elderly MM patients.

## 2. Frailty and Geriatric Assessment

Frailty is highly prevalent among elderly individuals and confers a high risk for falls, disability, hospitalization, and mortality [12]. Despite several positive reports regarding frail elderly MM patients, in reality, they often do not receive the appropriate treatment. As one of the underlying causes, the patient’s age is emphasized as a significant factor that influences the oncologist’s decision-making process [13]. While many frailty assessment tools are influenced by age [14], it is important to note that a discrepancy can arise between chronological age and biological age. Oncologists should not make treatment decisions for elderly MM patients based solely on their age. It is crucial to exercise caution in avoiding providing inadequate treatment. In the past, certain elderly MM patients may not have received adequate treatment based on their frailty [15].

However, given the availability of promising treatment approaches today, properly assessing frailty is essential in ensuring the delivery of the most appropriate treatment for each individual patient. As the development of treatment regimens for elderly MM patients continues to rapidly expand, the need for feasible and accurate frailty assessment in real-world clinical practice is becoming increasingly vital. This will ensure that the treatment provided to them in the future does not lead to undertreatment or overtreatment, given the potential proliferation of treatment options tailored for this population. Considering MM, several novel screening tools are currently recognized as a significant part of the geriatric assessment to evaluate frailty in elderly patients.

Typical examples encompass the International Myeloma Working Group (IMWG) frailty score [16], the FIRST simplified frailty scale [17], the revised Myeloma Comorbidity Index (R-MCI) [18], the UK Myeloma Research Alliance Myeloma Risk Profile (UKMRA MRP) [19], and the Mayo Risk Score [20] (Table 1). However, there are challenges in using, and depending upon, all of these tools, underscoring the need for further developments of more accurate assessment tools.

The IMWG developed a frailty index [16] that incorporates age, the Charlson comorbidity index (CCI) [21], the Katz ADL [22], and the Lawton instrumental ADL (IADL) [23]. The IMWG frailty scale classified patients as fit, intermediate, or frail, and these classifications were able to predict survival and the risk of toxicity from treatment in patients with NDMM. The IMWG frailty scale is indeed highly valuable; however, due to the somewhat time-consuming nature of assessing ADL and IADL, it might lack convenience in the context of routine clinical practice. Accordingly, Facon et al. [17] devised a simplified frailty scale using the Eastern Cooperative Oncology Group (ECOG) PS as a replacement for assessing ADL and IADL.

In a supplementary analysis of the Intergroupe Francophone du Myelome (IFM) FIRST trial, this tool was devised, and patients were categorized into two groups: non-frail and frail [17]. The analysis revealed that frail patients had inferior overall survival (OS) rates. The simplified frailty scale is a convenient and user-friendly tool in routine clinical practice. However, there are several limitations associated with it. Firstly, ECOG-PS is a subjective measure when compared to ADL and IADL, which introduces potential biases. Additionally, patients aged 80 or above automatically receive a score of 2, categorizing them as “frail” based solely on that criterion. It is important to note that there can be a discrepancy between biological age and chronological age, thus underscoring the need for caution [14,24].

The UKMRA MRP was developed by Cook et al. to stratify TIE mm patients [19]. This score incorporates the ECOG-PS, the International Staging System (ISS), age, and C-reactive protein concentration to predict survival outcomes. The risk score, however, does not consider patient comorbidities or functional testing. Furthermore, in 2017, the R-MCI was proposed as an improved version of the IMWG [18]. It has been shown to provide a clearer prognostic stratification. Factors such as age, physical assessment by Fried et al. [12], respiratory function, renal function, and the Karnofsky PS were adopted as contributing factors.

The cases were divided into three groups: fit, intermediate fitness, and frail. It was demonstrated that there was a nearly tenfold difference in the risk of death in the frail group. This index incorporates cytogenetic risks, and also takes disease-related factors into account [18]. In this classification, slightly younger patients (median age, 63 years) are targeted, and transplant-eligible MM patients are also included. As a result, this approach might be somewhat inappropriate when considering TIE mm patients.

The Mayo Risk Score evaluates three factors: N-terminal natriuretic peptide type B (NT-proBNP), the ECOG-PS, and age [20]. While NT-proBNP may assist in assessing the severity of cardiac amyloidosis [25,26], both the R-MCI and the Mayo Risk Score might lack a comprehensive assessment of the number of comorbidities for evaluating elderly MM patients who are likely to have multiple complications. In addition to the previously mentioned CCI [21], there are other methods for assessing comorbidities, such as the Hematopoietic Cell Transplantation-specific Comorbidity Index (HCT-CI) [27,28], the Kaplan–Feinstein Index (KFI) [29], and the Satariano Index (SI) [30] (Table 2). The CCI assesses 19 items, the HCT-CI evaluates 17 items, the KFI considers 12 items, and the SI includes 7 items for the evaluation of comorbidities. Not all of the items evaluated by these four comorbidity scores (CCI, HCT-CI, KFI, and SI) are significant for MM. However, according to Kleber et al., from the results of univariate and multivariate analyses, only three factors, namely the Karnofsky PS, moderate or severe lung disease, and the estimated glomerular filtration rate (eGFR) <30 mL/min/1.73 m^2^, were extracted as important prognostic determinants that could lead to decreased progression-free survival (PFS) and OS [31].

According to a report by Gregersen et al., MM patients have a higher incidence of comorbidities compared to the general population, particularly those aged 65 and older [32]. Comorbidities impact MM survival rates in various ways [32]. Not only do comorbidities independently increase the risk of death, but they can also amplify known risk factors of MM, such as severe infections and venous thromboembolism. Furthermore, comorbidities can influence the choice of MM treatment, potentially leading to dose reductions in medications and indirectly affecting prognosis [11]. Thus, when focusing on TIE mm patients, it is considered crucial to assess comorbidities comprehensively and appropriately. Additionally, it is vital to select chemotherapy regimens that include medications suitable for elderly MM patients with organ impairments, ensuring safe usage and directly positively impacting prognosis.

In clinical trials, eligibility criteria are generally established, and patients with a poor PS are often excluded. As an example, in the MAIA [5] and ALCYONE [33] trials, cases with a ECOG-PS ≥3, Creatinine clearance <30, psychiatric issues, active systemic infections, uncontrolled diabetes, and other coexisting conditions were excluded. The impact of not enrolling patients with a poor PS in clinical trials is believed to be substantial in real-world clinical practice. The simplified frailty scale [17] and the MRP [19] include the ECOG-PS as an assessment parameter, making it desirable to utilize such tools for building evidence concerning patients with a poor PS in practical clinical settings. When a patient has severe bone involvement as a complication, the ECOG-PS naturally deteriorates, and the patient may no longer qualify for enrollment in a clinical trial. However, if the bone involvement is related to MM, sufficient treatment should be introduced. It is important to consider the fact that the results of clinical trials may not directly apply to real-world clinical settings.

Furthermore, frailty is a dynamic condition, and it has been reported that the current state of frailty may predict outcomes better than the frailty state at diagnosis [34]. This underscores the need for proactive intervention, especially for patients with poor PS, as their current frailty status might be a more relevant factor to consider.

According to Jackson et al., regarding Myeloma XI (ISRCTN49407852) [35], it has been shown that as their age increases, the proportion of patients requiring dose adjustments of medications also increases, and the proportion of patients with treatment interruptions due to toxicity increases also. For elderly MM patients, dose adjustments to minimize toxicity and effectively manage treatment-related AEs are necessary. The currently ongoing FiTNEss trial (UK-MRA Myeloma XIV Trial) is a prospective study focusing on TIE-NDMM patients [36]. It aims to investigate whether or not determining the dosage of lenalidomide (LEN) and dexamethasone (DEX), based on the IMWG frailty score, can improve treatment continuation and reduce toxicity. That trial was designed with comprehensive selection criteria, allowing the enrollment of frail elderly MM patients who might not have been included in previous clinical trials. We eagerly await the results of this trial as these could illuminate the impact of this approach on patient outcomes.

The assessment items and screening tools for frailty and geriatric assessment are broad, and thus may require a considerable length of time, making their practical implementation in routine clinical practice difficult. The Geriatric 8 questionnaire [37], the Vulnerable Elders Survey 13 [38], and the Senior-Adult Oncology questionnaire [39] are tools that might allow for more efficient screening in a shorter amount of time. However, especially when targeting diverse elderly MM patients, it is essential to consider the perspective of taking ample time to thoroughly evaluate each patient for a thorough assessment.

If personnel shortage is a concern, investing in additional medical staff, even if it entails financial commitment, is considered a beneficial approach toward ensuring appropriate assessment and care provision [40]. While maximizing current resources, striking a balance with the number of feasible assessment items is crucial. The development of more efficient and innovative assessment tools and methods is anticipated. Furthermore, due to collaboration and a team-based approach among healthcare staff, the frailty assessment of elderly MM patients can be efficiently conducted, leading to the stratification of treatment strategies based on these results. Thus, tailored treatment aligned with each patient’s characteristics can be provided, in turn, fostering individualized care that accounts for frailty, and contributing to the enhancement of patients’ QOL and prognoses.

### 2.1. DARA-Containing Regimens for Frail Patients with TIE-MM

DARA is a humanized monoclonal antibody targeting a specific epitope on the CD38 protein, which is a transmembrane glycoprotein with ectoenzymatic activity and is predominantly present on the surface of plasma cells. Anti-CD38 antibodies such as DARA, which target CD38, have multifaceted mechanisms of action: (1) Fc (fragment crystallizable)-dependent immune effector mechanisms involving the antibody-dependent cell-mediated cytotoxicity, the antibody-dependent cellular phagocytosis, and the complement-dependent cytotoxicity, (2) direct effects including the induction of apoptosis and the inhibition of CD38 extracellular enzyme activity, and (3) immunomodulatory effects through the eradication of CD38-expressing immunosuppressive regulatory T and B cells and myeloid-derived suppressor cells [41]. After DARA administration, it is known that the MM cell surface CD38 expression levels rapidly decrease, at least in part, via trogocytosis [42]. However, due to the sustained immunomodulatory effects, gradual and deepening responses are expected over time [43,44]. Therefore, continuous treatment is necessary to achieve better efficacy.

In TIE-MM patients, initial treatment is crucial. The latest guidelines from the European Hematology Association and the European Society for Medical Oncology recommend standard treatments including DARA, LEN, and DEX (D-Rd); DARA, bortezomib (BTZ), melphalan (L-PAM), and prednisone (PSL) (D-VMP); and BTZ, LEN, and DEX (VRd). In cases where any of these three options are not available, either BTZ, L-PAM, and PSL (VMP), or LEN and DEX (Rd) are recommended [45]. However, it remains uncertain which of these three treatments, D-Rd, D-VMP, or VRd, is the optimal choice for the initial treatment of frail elderly MM patients.

According to Fonseca et al., it has been demonstrated that a DARA-containing regimen applied as the initial treatment leads to a longer median OS compared to introducing a DARA-containing regimen as a secondary treatment following VRd or Rd [46]. In the absence of head-to-head trials comparing D-Rd to VRd or to BTZ and DEX (Vd), the PEGASUS study compared PFS in patients treated with D-Rd in the MAIA trial and patients treated with common standard-of-care regimens from the Flatiron Health electronic health record-derived deidentified database [47]. Based on the indirect treatment comparisons, D-Rd demonstrated a significantly reduced risk of progression or death compared to both VRd and Vd.

It is generally known that the effectiveness of DARA tends to be better with higher levels of CD38 expression [44]. On the other hand, the decreased expression of CD38 in RRMM patients compared to NDMM patients has also been reported [48], which further supports the benefits of an early introduction of DARA. DARA is widely used in combination with other agents, not only in newly diagnosed cases, but also in relapsed/refractory cases [9,49,50,51]. Its high effectiveness has also been demonstrated. Here, we will summarize using DARA-containing regimens for initial treatment (Table 3) and subsequent therapy in frail elderly MM patients.

### 2.2. DARA-Containing Regimens for Frail Elderly Patients with TIE-NDMM

#### 2.2.1. The MAIA Trial

In the phase 3 MAIA trial, a total of 737 TIE-NDMM patients were randomly assigned to D-Rd (*n* = 368) or Rd (*n* = 369) [5]. Within the trial, 44% of the patients were aged 75 and older, indicating a relatively close resemblance to real-world clinical settings. After approximately 56 months of follow-up, a statistically significant and clinically meaningful improvement in the OS was observed with D-Rd versus Rd alone [6]. In addition, this clinical trial conducted post hoc analyses based on a frailty profile, using the simplified frailty scale [17]. The 36-month PFS rate was higher in the D-Rd cohort in all subgroups, with decreasing rates from fit to frail (total non-frail, the D-Rd group, 73.2% vs. the Rd group, 52.1%; frail, 61.5% vs. 39.5%, respectively) [52].

Therefore, although based on a retrospective assessment of frailty, D-Rd provided clinical benefits to patients with TIE-NDMM enrolled in the MAIA trial, regardless of their frailty status. In the total non-frail and frail subgroups classified by ISS I/II vs. III, respectively, the PFS benefit of D-Rd vs. Rd was maintained in most subgroups. However, the frail with ISS III subgroup was an exception; therefore, caution is needed when dealing with such populations [52]. 

In frail patients, the benefits of combining DARA are observed after approximately 12 months, but it is important to note that during the initial 12 months of treatment, the proportion of AEs as the reason for treatment interruption was notably high. This suggests that it is crucial to carefully manage AEs during this timeframe [52].

The frail subgroup had increased incidences of deaths and treatment-emergent AEs (TEAEs) resulting in death compared to all other frailty subgroups. In the D-Rd cohort, TEAEs resulting in death were observed in 3.6% of total non-frail patients and 11.9% of frail patients. Among frail patients, deaths occurring within 60 days of the first dose of the study treatment were reported in 6.0% of the D-Rd group and 3.6% of the Rd group, and within 90 days, in 7.7% of the D-Rd group and 4.2% of the Rd group. The primary cause of death in most of these frail patients was AEs (the D-Rd group, 92.3%; the Rd group, 85.7%). Overall, pneumonia was the most frequently observed treatment-emergent AE resulting in death in the frail subgroup (the D-Rd group, 1.2%; the Rd group, 1.8%) [52].

Serious AEs are not limited to pneumonia. Although higher rates of grade 3/4 neutropenia were observed with D-Rd in the frail subgroup compared to the total non-frail subgroup, these AEs were clinically manageable, and prompt granulocyte colony-stimulating factor (G-CSF) support should be provided for patients with neutropenia [55]. Additionally, LEN dose adjustments are also necessary to mitigate hematologic toxicities, including neutropenia. The median relative dose intensity (RDI) of LEN was lower with D-Rd when compared to Rd in all frailty subgroups, with the most significant disparity being observed in the frail subgroup. The median RDI of DARA was almost consistent across all frailty subgroups. Patients receiving DARA more frequently received a reduced initial dosage of LEN (<25 mg) across all frailty subgroups, with the highest occurrence being noted in the frail subgroup. G-CSF was predominantly employed in the frail subgroup and was more commonly administered alongside D-Rd than Rd across all frailty subgroups [52].

#### 2.2.2. The ALCYONE Trial

In the phase 3 ALCYONE trial, D-VMP vs. VMP significantly improved PFS and OS in TIE-NDMM patients [56]. As in the MAIA trial, this clinical trial conducted post hoc analyses based on frailty profiles, using the simplified frailty scale [17,53]. After 40.1 months of follow-up, which was the median amount of time in this study, non-frail patients had longer PFS and OS rates than frail patients; however, benefits of D-VMP vs. VMP were maintained across the subgroups: PFS in non-frail (median, 45.7 vs. 19.1 months), in frail (32.9 vs. 19.5 months); OS in non-frail (36-month rate, 83.6% vs. 74.5%), in frail (71.4% vs. 59.0%). During cycles 1 to 9, a lower proportion of D-VMP-treated patients discontinued treatment compared to VMP-treated patients in all frailty subgroups (total non-frail, 11.8% vs. 27.1%; frail, 28.8% vs. 41.7%). However, as frailty increases, cases also increase where treatment interruption occurs even in the D-VMP group. The ALCYONE trial revealed that D-VMP exhibited a greater PFS benefit when compared to VMP in both non-frail and frail patients. Interestingly, contrary to the results of the MAIA trial, the significance of D-VMP was demonstrated in frail ISS III patients. These findings are particularly noteworthy because they indicate that even in the most severely ill patients, specifically frail patients in the higher ISS disease stage category, D-VMP provides advantages in both PFS and OS compared to VMP. This result could potentially influence the decision-making process when considering using D-VMP or D-Rd in cases involving frail ISS III patients.

The two most common grade 3/4 TEAEs in all frailty subgroups with D-VMP and VMP were neutropenia and thrombocytopenia. Grade 3/4 peripheral sensory neuropathy (PSN) rates were low in all frailty subgroups with D-VMP and VMP. No patients in the D-VMP cohort discontinued treatment due to PSN. The low rates of treatment discontinuations due to PSN may be attributed to the use of a once-weekly BTZ dosing schedule during cycles 2 to 9. As seen in previous reports such as GIMEMA MM-03-05 [57] and GEM2005MAS65 [58], the once-weekly administration schedule of BTZ demonstrated a reduction in the incidence of grade 3/4 AEs and treatment discontinuation rates when compared to the twice-weekly administration schedule.

This highlights the potential for practicing continuous treatment while mitigating the occurrence of AEs through the appropriate adjustments of drug dosages. Therefore, further investigation into the optimal drug dosage for frailty is necessary. In that trial, both groups had a protocol for discontinuing VMP after the 10th cycle, but it became evident that there was an increase in cases of PD beyond that point. To address this issue, and to explore the balance between efficacy and safety, it is necessary to investigate the significance of continuing BTZ administration. It is noteworthy that ongoing clinical trials are currently addressing this matter [59]. Conversely, it is also important to mention that there are cases where patients do not experience relapse during DARA monotherapy. Therefore, determining what patients are suitable for transitioning to maintenance therapy and what patients require continued treatment remains a challenge for future investigations.

#### 2.2.3. The HOVON-143 Trial

The HOVON-143 trial, a phase 2 single-arm trial, enrolled sixty-five frail patients to receive nine cycles of DARA-ixazomib (IXA)-DEX induction, followed by DARA-IXA maintenance, and found an overall response rate (ORR) of 78% [54]. At the median follow-up point of 22.9 months, the median PFS was 13.8 months, and the 1-year OS was 78% (median OS, not reached). Although there was also an improvement in the QOL measure, during induction therapy, this trial reported the high rate of induction therapy discontinuation (51% of patients: PD, 19%; toxicity, 9%; death, 9%; noncompliance, 6%; and other causes, 8%) that negatively influenced PFS and OS rates. This study also reported differences in outcomes among patients classified as frail based upon age alone, or who were frail based on additional geriatric impairments as defined by the IMWG frailty score (median PFS 21.6 months for patients who were frail based on age >80 years alone vs. 10.1 months for patients who were frail based on age >80 and additional geriatric impairments). The key takeaway from these findings is that relying solely on age to determine frailty is not sufficient. It is important to accurately assess the patients’ overall conditions and to incorporate those evaluations into their treatment.

### 2.3. DARA-Containing Regimens for Frail Elderly Patients with TIE-RRMM

DARA is widely used in routine clinical practice for RRMM patients. In the phase 2 SIRIUS trial, patients with a median of five prior lines of therapy (95% of patients were refractory to both proteasome inhibitors [PIs] and immunomodulatory drugs) were treated with DARA monotherapy, resulting in an ORR of 29.2% [60]. Additionally, in the phase 1/2 study GEN503, the combination of DARA and Rd yielded a favorable ORR of 81% [61]. Here, we will summarize the data on representative DARA-containing regimens for frail patients.

#### 2.3.1. The CASTOR and POLLUX Trials

In the two phase 3 trials, CASTOR and POLLUX, the synergistic effects of adding DARA to the conventional standard treatments, Vd and Rd, were evaluated in RRMM patients. In both trials, the addition of DARA significantly improved ORR and PFS compared to the standard regimens, demonstrating substantial enhancements [9,49]. Subanalyses were conducted in both trials, evaluating the effectiveness and safety of combining DARA with standard treatment regimens in patients aged ≥75 years, as well as those aged 65–74 years.

In the CASTOR trial, among patients aged ≥75 years, DARA, BTZ, and DEX (D-Vd) exhibited a significant extension in PFS when compared to Vd (median, 17.9 months vs. 8.1 months), with an 18-month PFS rate of 45.8% in D-Vd and 0% in Vd. Among patients aged ≥75 years, 18 individuals (90.0%) in D-Vd and 26 individuals (74.3%) in Vd reported grade 3/4 TEAE. Particularly, thrombocytopenia was observed at frequencies of 45.0% in D-Vd and 37.1% in Vd among patients aged ≥75 years, making it the most common with grade 3/4 TEAE.

On the other hand, in the POLLUX trial, among patients aged ≥75 years, D-Rd showed a significant extension in PFS compared to Rd (median, 28.9 vs. 11.4 months), with 18-month PFS rates of 86.2% and 36.9%, respectively. Among patients aged ≥75 years, 25 individuals (86.2%) in D-Rd and 27 individuals (77.1%) in Rd had grade 3/4 TEAE. Among these, neutropenia was the most common grade 3/4 TEAE, occurring in 44.8% of patients in D-Rd and 31.4% in Rd among those aged ≥75 years.

However, these results have limitations. The proportion of patients aged ≥75 years in the CASTOR and POLLUX trials was relatively small (<15% of the total), and frailty was not assessed in these trials. Nonetheless, the subanalyses of the POLLUX and CASTOR trials indicated that the combination of DARA with Rd or with Vd is not adversely affected by age [62]. Additionally, introducing DARA at an early treatment stage seems to provide stronger benefits [10,63].

#### 2.3.2. The CANDOR Trial

The efficacy and safety of treatment for frail RRMM patients were evaluated in three phase 3 trials [64]: ASPIRE (twice-weekly carfilzomib [CFZ] of 27 mg/m^2^ and Rd [KRd27] vs. Rd) [65], ENDEAVOR (twice-weekly CFZ of 56 mg/m^2^ and DEX [Kd56] vs. Vd) [66], and A.R.R.O.W. (once-weekly CFZ [70 mg/m^2^]-DEX [Kd70] vs. CFZ [27 mg/m^2^]-DEX [Kd27]) [67]. These trials employed a simplified frailty scale to assess frailty, and the effectiveness and safety of the treatment regimens were evaluated in the frailty subgroups. Notably, in all three trials, consistent efficacy and safety were observed across the frailty subgroups, irrespective of frailty status.

Even though the combination of DARA with Kd56 (D-Kd) was investigated in the CANDOR trial [51,68,69], data on patients classified as frail based on the simplified frailty scale [17] were reported by Quach et al. [70]. The results aligned with previous investigations [68], showing that the benefits of efficacy and safety for D-Kd compared to Kd56 were maintained within the entire frail subgroup, with observed PFS benefits and no increase in toxicity. Although cardiac toxicity is a concern with CFZ, its frequency appears to decrease when combined with DARA (grade 3 or higher cardiac failure: D-Kd 3.9%, Kd 8.5%) [51]. This trend also holds true for frail patients (cardiac failure: non-frail D-Kd 3.2%, Kd 6.4%; frail D-Kd 5.2%, Kd 15%) [70]. The proper assessment of cardiac function during screening and cautious management after treatment initiation can maximize the therapeutic regimen’s potential benefits for frail patients. Additionally, D-Kd therapy is particularly advantageous for elderly MM patients, where dosing schedules are critical.

According to the phase 2 PLEIADES trial and phase 1b EQUULEUS trial, combining subcutaneous DARA (DARA-SC) and intravenous DARA (DARA-IV) with once-weekly Kd70 resulted in good tolerability and deep responses in RRMM patients [71,72,73]. However, limitations of the two trials, PLEIADES and EQUULEUS, include small sample sizes and a lack of comparator arms. Larger-scale trials, focusing especially on frail RRMM patients, are needed for further validation.

#### 2.3.3. The APOLLO Trial

The APOLLO trial investigated the efficacy of the combination therapy of DARA, pomalidomide, and DEX (D-Pd) for patients with RRMM [50,74]. At a median follow-up of 16.9 months, D-Pd exhibited superior PFS when compared to Pd alone (median, 12.4 months vs. 6.9 months) [50]. In the final report, at a median follow-up of 39.6 months, the D-Pd demonstrated a longer median OS when compared to Pd alone (34.4 months vs. 23.7 months), exhibiting its efficacy [74].

However, similarly to the CANDOR trial, the proportion of elderly patients aged ≥75 years was limited, warranting the accumulation of data for the frail elderly MM population, especially the “truly frail” patients.

## 3. The Effect of DARA-Containing Regimens on the QOL in Elderly MM Patients with Frailty

Among cancer survivors, MM patients have shown low QOL scores, emphasizing the significance of continuous QOL assessment and interventions for QOL enhancement [3,75,76]. Various factors influencing QOL are recognized, including: (1) patient-reported symptoms such as anxiety and pain, (2) complications, (3) treatment-related toxicities, and (4) treatment response [75,77,78,79,80,81,82]. Particularly, a positive treatment response (specifically partial response [PR] or better) is anticipated to alleviate bone pain, anemia symptoms, and organ impairments, leading to QOL improvements, alongside enhanced physical strength and activities [83].

Furthermore, while QOL improvements are more apparent in MM patients under 70 years of age compared to those older than 70 years [83], frailty is often linked with reduced QOL [75]. Therefore, especially in frail elderly patients, the goal must include achieving favorable treatment outcomes, but also addressing bone pain and anemia symptoms experienced at the onset, and further striving to improve the overall QOL. Sustaining QOL facilitates ongoing treatment, enabling longer-term disease control, and potentially fostering better overall prognosis. Here, we contemplate the impact of DARA regimens on QOL in frail elderly MM patients.

At the time of its development, DARA was administered intravenously. However, it had been previously reported that for biologic agents used in cancer treatment, subcutaneous administration had positive effects on the patients’ health-related QOL (HRQOL), healthcare resource utilization, and economic aspects compared to intravenous administration [84,85]. In the phase 2 PLEIADES trial, the clinical efficacy of DARA-SC added to the conventional standard treatment was demonstrated to be equivalent to DARA-IV [71]. Furthermore, in the phase 3 COLUMBA trial, DARA-SC not only showed comparable efficacy to DARA-IV, but also had a lower incidence of infusion-related reactions (IRRs) (12.7% vs. 34.5%, respectively). Additionally, the administration time was significantly shortened (3–5 min vs. 3–7 h), leading to a marked improvement in QOL [86,87,88]. The switch from DARA-IV to DARA-SC has proven beneficial for both patients and healthcare providers in real-world clinical settings. It simplifies treatment, reduces the burden on hospitals, and enhances QOL [89]. Notably, DARA-SC is likely to be safely used for patients at a higher risk of complications due to volume overload, such as frail patients with comorbidities like chronic heart failure or end-stage renal disease requiring dialysis.

In the VISTA trial [90], the adverse event profile associated with BTZ suggests a decline in QOL during the twice-weekly administration of BTZ. However, reducing the dosing frequency of BTZ to once a week can decrease the frequency of AEs and avoid QOL deterioration, thereby enabling continued treatment [91,92].

In the ALCYONE trial, a protocol was designed from the second cycle onwards to administer BTZ once a week [33]. QOL was assessed in this trial as well, and data supporting the previously reported clinical efficacy benefits were obtained, with both the D-VMP and VMP groups maintaining functional and health statuses [93]. Notably, in the subgroup analyses based on age and ECOG-PS, an increasing trend in the improvement of HRQOL was observed for all subgroups in terms of the European Organization for Research and Treatment of Cancer Quality of Life Questionnaire Core 30-item (EORTC QLQ-C30) Global health status (GHS), physical functioning, pain, and fatigue scores. Additionally, time to deterioration in EORTC QLQ-C30 GHS was significantly longer for patients with a complete response when compared to patients with a very good partial response (VGPR)/PR (HR = 0.72, *p* = 0.025), while patients with a stable disease showed significantly shorter time (HR = 1.75, *p* = 0.008).

Similarly, patients who achieved minimal residual disease (MRD) negative status showed significant improvements in the EORTC QLQ-C30 GHS and pain compared to the MRD-positive patients (HR = 0.70, *p* = 0.042 and HR = 0.60, *p* = 0.002) [93]. These findings suggest that as the response depth increases, the QOL improves, and the significance of combining DARA with VMP from both an efficacy and QOL perspective was demonstrated, regardless of age.

On the other hand, while the FIRST trial demonstrated the superiority of Rd over MPT [94,95], the Rd not only delayed the PD compared to the MPT, but also showed clinically meaningful improvements in HRQOL [96]. Subgroup analyses based on age, for patients aged ≤75 or >75 years, were conducted, revealing that changes in the “Side Effects of Treatment” in the EORTC QLQ Multiple Myeloma Module 20-item were statistically significantly favorable in the Rd when compared to the MPT for all age groups (*p* < 0.05). Additionally, in patients aged ≥75 years, both “Pain” and “Disease Symptoms” showed significant improvements in both treatment groups, but the Rd had significantly better scores in the EuroQol 5-dimensional descriptive system Healthy Utility and Physical Functioning when compared to the MPT group (*p* < 0.05). 

However, the MAIA trial yielded results that surpassed the benefits of Rd. In the MAIA study, the D-Rd demonstrated faster and sustained improvement in the HRQOL measures compared with the Rd in patients with TIE-NDMM [97]. Improvements in GHS scores were observed from cycle 3 regardless of age, and, a majority of the time, points showed greater improvements in the D-Rd than that in the Rd. Furthermore, irrespective of age, the D-Rd reported significant early and substantial pain relief compared to that in the Rd [98]. In essence, even in elderly MM patients, combining DARA with Rd can yield better efficacy and improvement in QOL. The HOVON-143 trial, a phase 2 trial targeting frail patients, not only demonstrated a high response rate but also resulted in improvements in HRQOL [54]. Impressively, these meaningful improvements were already observed at the end of cycle 3 after the treatment initiation. However, issues of treatment interruption due to toxicity and early death remained, emphasizing the importance of balancing effectiveness, QOL, and AE management, particularly in frail elderly MM patients.

Similar findings have been demonstrated in the context of RRMM patients. In the CASTOR trial, during the initial eight cycles, there were no changes in patients’ HRQOL in either of the treatment groups. However, patients receiving long-term DARA therapy reported improvements in GHS and pain following this point [99]. Furthermore, a post hoc analysis stratified by age (<65 years vs. ≥65 years) was conducted, showing that the benefits of incorporating DARA were observed regardless of age. In the POLLUX trial, the baseline HRQOL was sustained with long-term D-Rd treatment, aligning with the sustained and significant PFS extension effect of D-Rd. This supported the use of D-Rd for RRMM patients irrespective of age. However, although the D-Rd showed statistically significant larger average changes from the baseline in EORTC QLQ-C30 GHS, physical functioning, and pain scores than the Rd at multiple time points, the magnitude of change was modest, and a meaningful impact on the HRQOL was not indicated [100].

In the CANDOR trial, among the prespecified subgroups, no significant differences were observed between the treatment groups; however, patients aged ≥75 years showed a trend of improvement in GHS/QOL scores in the Kd (mean difference 3.97) [101]. Yet, due to the limited proportion of patients aged ≥75 years, larger-scale validation is deemed necessary, and adjustments in CFZ administration schedule may be necessary to maintain QOL in older adults.

Additionally, the APOLLO trial suggested that patients aged ≥65 years had the highest likelihood of exhibiting significant favorable differences in the least squares mean change from the baseline in GHS, pain, physical functioning, and emotional functioning, favoring D-Pd [102]. In any case, the benefits of DARA-containing regimens in the context of RRMM patients are supported from the perspective of their impact on QOL. Therefore, it is advisable to consider their use. Undertaking a larger and more comprehensive examination and analysis focusing on frail RRMM patients is considered an urgent priority.

In frail elderly patients, seeking effectiveness alone is not sufficient, as treatment goals can vary for each individual patient. Therefore, it is important to engage in thorough discussions with the patient’s themselves and their families, determining treatment goals. However, by incorporating DARA into conventional standard treatments like VMP or Rd, not only can high efficacy be expected, but also significant benefits from a QOL perspective, particularly in frail elderly patients. DARA-containing regimens offer the advantage of aiming for and maintaining MRD negativity compared to regimens without DARA [6,10,50,56,63,69]. Therefore, these regimens can lead to improvements in QOL, making them a mutually beneficial solution.

## 4. Supportive Therapy

As previously mentioned, DARA-containing regimens are excellent in terms of both efficacy and QOL for frail elderly MM patients with NDMM and RRMM. While the impact of ISS [103] and chromosomal abnormalities [104,105] on prognosis is widely recognized, the Myeloma XI trial demonstrated that PS has a significant influence on OS, particularly in elderly MM patients [106]. Efforts are needed to prevent and improve worsening PSs and frailty after treatment initiation. To achieve this, it is crucial not only to strive for maintaining efficacy and QOL, but also to manage bone lesions, organ dysfunctions in the kidneys and heart, and treatment-related AEs. In this context, we will discuss the expected impact of DARA on organ dysfunctions and provide insights into common AEs anticipated with various treatment regimens and strategies to address them.

### 4.1. Management of MM-Related Bone Disease

The incidence of lytic bone lesions in NDMM patients is known to reach approximately 80% [107]. The presence of lytic bone lesions increases the risk of skeletal-related events. This could potentially impact not only the QOL, but also survival rates due to pathological fractures and spinal cord compression [108,109,110]. The primary cause of decreased QOL is often attributed to skeletal-related events. Managing these bone lesions requires the effective control of MM itself, collaboration with orthopedic surgeons to stabilize fractures, and appropriate supportive therapy for pain control.

To enhance the improvement of poor PS or frailty, it may be worth considering not only chemotherapy for MM, but also exploring the potential for improvement through rehabilitation. As an objective alternative to PS, gait speed could be considered. It has been noted that with each decrease of 0.1 m per second in gait speed, there is an increase in mortality and unplanned hospitalizations [111]. Gait speed has the potential to be an indicator helping to predict outcomes for patients with hematologic malignancies, including those with MM aged 75 years and older. While gait speed can help predict survival, it may not directly quantify the recommended treatment intensity. By incorporating gait speed as one of the indicators during treatment decision making, there is a potential for an improved selection of optimal treatment approaches [112].

On the other hand, osteocytes undergo bone formation through localized mechanical loading stimuli. However, in physical states where there is no mechanical load due to factors such as bedridden conditions, increased expression of receptor activator of the nuclear factor-kappa B ligand leads to enhanced osteoclast formation and bone resorption [113,114], i.e., rehabilitation aimed at avoiding a decline in ADL and PS could potentially offer benefits from a bone remodeling perspective as well. In this context, the REBUILD study, a prospective, open-label, phase 2 trial, investigated the impact of DARA on bone remodeling in RRMM patients [115]. DARA was shown to induce bone formation and improve bone turnover by reducing the inhibition of osteoblasts. In fact, considering the improvements in symptoms such as bone pain observed in the MAIA trial without the use of PIs [98], it may not be absolutely necessary to use PIs in cases with bone lesions.

### 4.2. Management of Renal Impairment

The prognosis of MM patients with renal impairment (RI) is often worse than those without it [116]. Data on the use of DARA in patients with severe RI are limited, but the phase 2 DERA trial (NCT03450057) provides valuable insights [117]. In this trial, 38 patients with severe RI, defined as an eGFR below 30 mL/min/1.73 m^2^, were enrolled, including 17 (44.7%) who were on dialysis. Patients with such severe RI are generally excluded from clinical trials. Both dialysis and non-dialysis patients showed consistent efficacy in terms of PFS and ORR. The median PFS was 11.8 months, with 2.8 months in dialysis patients and 13.3 months in non-dialysis patients (*p* = 0.358). The ORR was 47.4% with PR at 13.2% and VGPR at 34.2%. Among dialysis patients, the ORR was 47.1% (PR, 17.6%; VGPR, 29.4%), and, among non-dialysis patients, it was 47.6% (PR, 9.5%; VGPR, 38.1%). The renal response rate (PR renal or higher) was 18.4%, and one dialysis patient was able to discontinue dialysis.

As reported by Kuzume et al. [118] and Cejalvo et al. [119], DARA can be safely administered to MM patients with severe RI, and even the possibility of discontinuing dialysis has been demonstrated. In summary, the efficacy of DARA has been demonstrated in patients with severe RI and those requiring dialysis. Given that RI is not uncommonly due to causes other than aging in the elderly, kidney biopsy is necessary to investigate the underlying cause, although it is often challenging to perform. In such situations, the proper evaluation of urine protein fractions is important for making a clinical estimation [120]. Moreover, cases with improved renal function have shown improved prognosis [116], particularly for those with cast nephropathy, where the rapid reduction of involved free light chains can predict renal prognosis improvement [121]. Therefore, the ability to initiate early treatment intervention promptly is crucial. To achieve this, rather prologing treatment due to RI, it is advisable to be more proactive in treatment intervention [122].

### 4.3. Management of Cardiac Complications

Elderly individuals are known to have a higher prevalence of cardiovascular diseases, such as coronary artery disease and heart failure [123,124]. Among the treatment options for MM, CFZ in particular is expected to have a high efficacy, but concerns about cardiotoxicity arise in routine clinical practice. However, there have been several reports suggesting that the combination of CFZ with DARA may attenuate these concerns [68,125]. This is welcome news for elderly MM patients who often have multiple comorbidities. Furthermore, DARA can be administered subcutaneously without concerns of dose overload. CD38 is expressed not only in myeloma cells, but also in various normal tissues, particularly in endothelial cells, where its high expression leads to impaired endothelial function due to reduced reoxygenation after ischemia [126]. The inhibition of CD38 via the administration of DARA may mitigate post-ischemic endothelial damage and exert a cardioprotective effect [127], although further investigation is necessary for validation.

### 4.4. Management of Infectious Diseases

Patients with MM have been reported to have a higher risk of developing bacterial and viral infections when compared to healthy individuals [128]. In elderly MM patients, survival rates decrease due to the treatment interruptions caused by AEs during initial therapy [11]. Infections are considered one of the major causes of early mortality [129]. One important aspect of avoiding infections is the management of neutropenia. Adjusting the dosages of drugs that may cause neutropenia, such as LEN, for each case, and actively supporting with G-CSF are crucial steps [55]. However, for elderly frail MM patients, frequent G-CSF support is considered limited, as it often requires clinic visits. While securing caregivers is important, it is also crucial to consider enhancing G-CSF administration support via home visit medical care. By focusing on building close collaborations with regional healthcare and home visit medical care providers, long-term continuous treatment becomes possible. 

On the other hand, considering blood toxicity, adjusting the dosage of LEN is also necessary. The European Myeloma Network provides recommended drug dosages based on frailty categories [130]. However, the reduction criteria for drug dosage mentioned that there are not evidence-based, but rather based on experts’ empirical knowledge. An exploratory phase 2 clinical trial (NCT04223661) was under consideration to determine whether or not adjusting the dosage of LEN based on a patient’s frailty would be effective in treatments with the combination of DARA and DEX (D-Rd lite). Unfortunately, this trial was discontinued midway, but its design involved initiating LEN at a starting dose of 10 mg and then escalating it to 15 mg for frail patients. Through the continued verification and accumulation of evidence, such investigations could further enhance the benefits of D-Rd therapy. In the MAIA and ALCYONE trials, an increased frequency of early infections, particularly pneumonia, shortly after treatment initiation has been observed (≤90 days in the MAIA trial) [56]. The prophylactic administration of antibiotics such as levofloxacin to patients considered at high risk of infections [131,132] can prevent the occurrence of early infections after treatment initiation.

There are also infectious diseases that can be prevented through vaccination. Firstly, we will discuss the coronavirus disease 2019 (COVID-19). Patients with hematologic disorders are in the high-risk group for COVID-19, and, among them, MM patients, especially the elderly, require special attention [133]. The importance of vaccination is evident. Several studies on antibody levels following vaccination in MM patients have reported factors contributing to lower positive rates and decreased antibody levels of IgG (immunoglobulin) antibodies, such as advanced age, uncontrolled disease status, multiple comorbidities, low levels of normal immunoglobulins, and lymphocyte reduction [134,135]. Additionally, patients undergoing DARA-containing regimens have been shown to have significantly lower antibody levels when compared to untreated or observed patients [134,135,136,137]. Particularly for frail patients during the COVID-19 pandemic, it is wise to take preventive measures such as wearing masks, social distancing, avoiding crowded places, and recommending vaccination [133].

Regarding elderly, herpes zoster patients, the incidence is approximately 2.0 to 4.6 cases per 1000 person years, increasing with age to 10.0 to 12.8 cases per 1000 person years for individuals ≥80 years of age [138]. Similarly, the incidence of post-herpetic neuralgia also increases with age [139]. Because post-herpetic neuralgia impairs patients’ QOL, prevention is necessary. Patients treated with PIs [140], DARA [141], autologous stem cell transplant following high-dose L-PAM, and high-dose corticosteroids are at an increased risk of varicella-zoster virus reactivation. Adopting a proactive stance in recommending the use of the recombinant varicella-zoster virus glycoprotein E vaccine is reasonable for most MM patients [142]. Patients should receive two doses of the vaccine at intervals of 2 to 6 months, combined with conventional preventive measures using acyclovir or valacyclovir, in order to achieve further risk reduction [143]. This is particularly important for patients receiving PIs or anti-CD38 antibodies.

In DARA-containing regimens, there is a potential for various infections due to hypogammaglobulinemia. For MM patients facing this risk, immunoglobulin replacement therapy may be considered. According to Lancman et al., a significant reduction in the incidence of infections has been reported in patients with MM through the use of immunoglobulin replacement therapy [144]. However, due to the lack of consensus regarding the specific values and frequency of initiating immunoglobulin replacement, it is necessary to individually consider each patient’s case in practical settings.

Nevertheless, particularly in elderly MM patients, infections can impede treatment and pose a threat to overall prognosis. Therefore, considering the impact of infections, it would likely be worthwhile to provide immunoglobulin replacement therapy as a means of preventing such challenges, which would hopefully ensure better outcomes.

### 4.5. Management of Steroid Usage

Compared to lower doses, higher doses of DEX have been reported to increase the risk of non-hematologic AEs such as infections [145]. Furthermore, the long-term use of steroids can lead to cataracts. In the MAIA trial [6], a protocol involving continuous steroid use resulted in a higher frequency of cataracts. However, the ALCYONE trial [53] and the HOVON 143 trial [54] did not show a significant increase in cataract frequency, suggesting that steroid administration should be kept to a minimum.

In recent years, there has been active debate regarding the timing of steroid discontinuation. The phase 3 randomized controlled trial RV-MM-PI-0752, conducted in Italy, included intermediate-risk NDMM patients based on the IMWG frailty scale [146]. After receiving nine cycles of Rd, a group that discontinued DEX was compared to a group that continued DEX. The key endpoint, event-free survival, indicated that the discontinued DEX group had a median of 10.4 months compared to 6.9 months in the continued DEX group (*p* = 0.02), suggesting that reducing steroid use resulted in fewer events. Corticosteroid-induced immunosuppression may potentially reduce the efficacy of immunotherapy [147].

In DARA-containing regimens, corticosteroids are used to mitigate the risk of IRRs associated with DARA administration. However, limiting the use of steroids might be advisable to maximize the immunomodulatory effect of DARA. The PAVO part 3 trial evaluated three corticosteroid tapering schedules before and after DARA-SC administration [148]. Notably, the PAVO trial is a phase 1b, open-label, multicenter, dose-finding, proof-of-concept trial [149,150]. Results showed that corticosteroid tapering did not compromise the efficacy of DARA-SC monotherapy, and patients who received DARA-SC with or without corticosteroid tapering achieved similar effectiveness. Such perspectives are especially important for frail elderly MM patients, warranting further clinical trials. The presently ongoing phase 3 IFM2017-03 trial (NCT03993912) compares DARA-LEN therapy without prolonged DEX to Rd therapy in frail NDMM patients to analyze response rates and safety. According to interim analysis, a DEX-sparing regimen combining DARA and LEN demonstrates deep and rapid responses and a favorable safety profile, but longer-term observations are warranted [151]. Additionally, the phase 2 IFM 2018-02 trial (NCT03757221) is investigating the safety of steroid-free DARA-IXA treatment in elderly RRMM patients [152]. Careful consideration of the results from these clinical trials is necessary to determine appropriate steroid usage for frail patients.

## 5. Future Perspectives

The currently ongoing CEPHEUS trial (NCT03652064) is a phase 3 trial related to DARA [153]. CEPHEUS will evaluate participants with NDMM for whom hematopoietic stem cell transplant is not planned as an initial therapy. The purpose of this trial is to determine if the addition of DARA to VRd will improve the overall MRD negativity rate when compared to VRd alone. This is a highly promising treatment regimen, the results of which are eagerly awaited. However, it should be noted that this trial has set the exclusion criteria of a frailty index of ≥2 according to the myeloma geriatric assessment score; therefore, verification from this perspective is expected to remain a future challenge. On the other hand, in recent years, the second-generation anti-CD38 monoclonal antibody, isatuximab (ISA), has been approved. DARA and ISA both have similarities and differences in their mechanisms of action due to binding to distinct regions of the CD38 molecule [154]. Currently, there are two ongoing phase 3 clinical trials. Specifically, the IMROZ trial (NCT03319667) is comparing the efficacy of ISA-VRd and VRd in TIE-NDMM patients, with PFS being the primary endpoint. The BENEFIT trial (NCT04751877) will compare the efficacy of ISA-VRd and ISA-Rd in TIE-NDMM patients aged 65–79 years, with the primary endpoint being the MRD negativity rate at 18 months. The phase 2 GMMG-CONCEPT trial (NCT03104842) is investigating the four-drug combination regimen of ISA-KRd as a primary treatment for high-risk elderly MM patients [155].

In the future, as a greater variety of treatments become available for TIE-MM patients, the choice between using DARA or ISA, opting for a three- or four-drug combination regimen, will undoubtedly become more complex. By reassessing the frailty assessment tool for TIE-MM patients and further enhancing it, it will become possible to formulate treatment strategies tailored to each individual patient’s frailty, i.e., accurately stratifying the diverse group of elderly MM patients based on their frailty will aid in treatment decision-making, avoiding both undertreatment and overtreatment, and providing each patient with the most suitable treatment. Therefore, the implementation of clinical trials considering such factors is crucial. As healthcare professionals, we are responsible for the enhancement of treatments, resulting in optimal outcomes, and striving to ensure that the treatments and procedures are satisfying for all of our frail elderly MM patients.

## 6. Conclusions

While significant progress has been made in the treatment of MM, the treatment outcomes for elderly patients, especially those who are frail, remain insufficient. DARA-containing regimens have demonstrated effectiveness and safety for MM patients, including frail elderly patients, and their benefits have been shown in terms of QOL as well. In real-world clinical practice, there is a diverse population of elderly MM patients that surpasses what is seen in clinical trials.

Moreover, due to certain limitations in the present frailty assessment tools, there is an urgent need to develop highly accurate screening tools that comprehensively evaluate frailty, i.e., the evidence for DARA-containing regimens in truly frail elderly MM patients is still considered inadequate. However, it is important to avoid judging frailty solely by age, and, as healthcare providers, we have a responsibility to creatively and thoroughly consider whether the benefits of DARA-containing regimens can be extended to patients with organ impairments and/or low ADL levels.

In the future, an expansion of treatment options for TIE-MM patients is expected. However, if frail elderly MM patients can be adequately assessed, categorized, and administered treatment based on their frailty, the treatment outcomes for TIE-MM patients could be improved, leading to more satisfaction.

## Figures and Tables

**Table 1:** Frailty scoring tools.

Tools	Variables	Stratification and Scores (*n*) for Each Variable	Patient Status and Total Score
IMWG frailty scale [16]	1. Age	≤75 (0), 76–80 (1), >80 (2)	Fit: 0 Intermediate fitness: 1 Frail: ≥2
	2. CCI	≤1 (0), ≥2 (1)	
	3. ADL	>4 (0), ≤4 (1)	
	4. IADL	>5 (0), ≤5 (1)	
Simplified frailty scale [17]	1. Age	≤75 (0), 76–80 (1), >80 (2)	Non-frail: 0–1 Frail: ≥2
	2. CCI	≤1 (0), >1 (1)	
	3. ECOG-PS	0 (0), 1 (1), ≥2 (2)	
R-MCI [18]	1. Age	<60 (0), 60–69 (1), ≥70 (2)	Fit: ≤3 Intermediate fitness: 4–6 Frail: >6
	2. Renal function (eGFR_MDRD_)	≥60 (0), <60 (1)	
	3. Pulmonary function	No/mild (0), Moderate/severe (1)	
	4. Karnofsky PS	100% (0), 80–90% (2), ≤70% (3)	
	5. Frailty status	No/mild (0), Moderate/severe (1)	
	6. Cytogenetics	Favorable/unavailable (0), Unfavorable (1)	
UKMRA MRP [19]	1. Age	a: (Ag − 74.4) × 0.089 ÷ 5.40	(a + b + c + d = e) Low risk: e < –0.256 Medium risk: –0.256 ≤ e ≤ –0.0283 High risk: e > –0.0283
	2. WHO PS (score)	b: 0 (–0.398), 1 (–0.199), 2 (0.000), 3 (0.199), 4 (0.397)	
	3. ISS stage	c: I (–0.212), II (0), III (0.212)	
	4. CRP levels (mg/L)	d: (log_e_ [CRP + 1] − 2.08) × 0.035 ÷ 1.11	
Mayo Risk Score [20]	1. Age	<70 (0), ≥70 (1)	Stage I: 0 Stage II: 1 Stage III: 2 Stage IV: 3
	2. ECOG-PS	<2 (0), ≥2 (1)	
	3. NT-proBNP levels (ng/L)	<300 (0), ≥300 (1)	

IMWG, International Myeloma Working Group; R-MCI, Revised Myeloma Comorbidity Index; UKMRA MRP, UK Myeloma Research Alliance Myeloma Risk Profile; CCI, Charlson comorbidity index; ADL, activities of daily living; IADL, instrumental ADL; ECOG, Eastern Cooperative Oncology Group; WHO, World Health Organization; PS, performance status; eGFRMDRD, estimated glomerular filtration rate by modification of diet in renal disease; CRP, C-reactive protein; ISS, international staging system; NT-proBNP, N-terminal natriuretic peptide type B.

**Table 2:** Methods for assessing comorbidities.

CCI [21]	HCT-CI [27,28]	KFI [29]	SI [30]
Myocardial infarction Congestive heart failure Peripheral vascular disease Cerebrovascular disease Dementia Chronic pulmonary disease Connective tissue disease Ulcer disease Mild liver disease Diabetes Hemiplegia Moderate/severe renal disease Diabetes with end organ damage Any tumor without metastasis Leukemia Lymphoma Moderate/severe liver disease Metastatic solid tumor AIDS	Arrhythmia Cardiac Inflammatory bowel disease Diabetes Cerebrovascular disease Psychiatric disturbance Hepatic, mild Obesity Infection Rheumatologic Peptic ulcer Moderate/severe renal disease Moderate pulmonary Prior solid tumor Heart valve disease Severe pulmonary Moderate/severe hepatic disease	Hypertension Cardiac Cerebral or psychic Respiratory Renal Hepatic Gastro-intestinal Peripheral vascular Malignancy Locomotor impairment (regardless of cause) Alcoholism Miscellaneous	Heart disease (not myocardial infarction) Gallbladder condition Diabetes Cancer (not breast cancer) Respiratory condition Liver condition Myocardial infarction

CCI, Charlson comorbidity index; HCT-CI, Hematopoietic cell transplantation-specific comorbidity index; KFI, Kaplan–Feinstein index; SI, Satariano index.

**Table 3:** Summary of DARA-containing regimens as initial treatment for frail elderly MM patients.

	MAIA Trial [52]		ALCYONE Trial [53]		HOVON-143 Trial [54]
	DARA + LEN + DEX		DARA + BTZ + L-PAM + PSL		DARA + IXA + DEX
	Non-Frail (*n* = 196)	Frail (*n* = 172)	Non-Frail (*n* = 187)	Frail (*n* = 163)	Frail (*n* = 65)
Age, median (range), yr	71.0 (50–80)	77.0 (57–90)	70.0 (52–80)	74.0 (40–93)	81 (70–92)
<65, *n* (%)	2 (1.0)	2 (1.2)	13 (7.0)	23 (14.1)	ND
65–<70 yr, *n* (%)	56 (28.6)	18 (10.5)	150 (80.2) (65–<75 yr)	60 (36.8) (65–<75 yr)	ND
70–<75 yr, *n* (%)	98 (50.0)	32 (18.6)			ND
≥75 yr, *n* (%)	40 (20.4)	120 (69.8)	24 (12.8)	80 (49.1)	ND
≥80 yr, *n* (%)	6 (3.1)	60 (34.9)	1 (0.5)	32 (19.6)	33 (51) (>80 yr)
ECOG-PS					
0, *n* (%)	107 (54.6)	20 (11.6)	66 (35.3)	12 (7.4)	9 (13.8)
1, *n* (%)	89 (45.4)	89 (51.7)	121 (64.7)	61 (37.4)	29 (44.6)
2, *n* (%)	0 (0.0)	61 (16.6)	0 (0.0)	90 (55.2)	20 (30.8)
>2, *n* (%)	0 (0.0)	2 (0.5)	0 (0.0)	0 (0.0)	5 (7.7)
Unknown, *n* (%)	0 (0.0)	0 (0.0)	0 (0.0)	0 (0.0)	2 (3.1)
Creatinine clearance, mL/min					
≥60, *n* (%)	133 (67.9)	73 (42.4)	121 (64.7)	79 (48.5)	ND
30–<60, *n* (%)	63 (32.1)	92 (53.5)	65 (34.8)	82 (50.3)	ND
<30, *n* (%)	0 (0.0)	7 (4.1)	1 (0.5)	2 (1.2)	ND
Efficacy					
Median follow-up, months	36.4 mo	36.4 mo	40.1 mo	40.1 mo	22.9 mo
ORR, *n* (%)	192 (98.0)	150 (87.2)	174 (93.0)	144 (88.3)	51 (78.5)
VGPR or better, *n* (%)	167 (85.2)	128 (74.4)	138 (73.8)	117 (71.8)	23 (35.4)
PR, *n* (%)	25 (12.8)	22 (12.8)	ND	ND	28 (43.1)
MRD negative (10–5), *n* (%)	65 (33.2)	41 (23.8)	52 (27.8)	47 (28.8)	4 (6.2)
Median PFS, months	NR	NR	45.7 mo	32.9 mo	13.8 mo
Median DoT, months	33.6 mo	31.1 mo	36.4 mo	24.7 mo	ND
Safety					
Infections, Grade 3 or worse, *n* (%)	62 (31.6)	70 (41.7)	44 (23.7)	48 (30.0)	16 (24.6)
Pneumonia, Grade 3 or worse, *n* (%)	20 (10.2)	33 (19.6)	22 (11.8)	23 (14.4)	ND
TEAEs with outcomes of death, *n* (%)	7 (3.6)	20 (11.9)	7 (3.8)	17 (10.6)	0 (0.0)
Discontinuation due to PD, *n* (%)	39 (19.9)	32 (18.6)	9 (4.8)	14 (8.8)	12 (18.5)
Discontinuation due to AEs, *n* (%)	14 (7.1)	17 (9.9)	7 (3.8)	11 (6.9)	6 (9.2)
Discontinuation due to noncompliance, *n* (%)	6 (3.1)	8 (4.7)	1 (0.5)	9 (5.6)	4 (6.2)

DARA, daratumumab; LEN, lenalidomide; DEX, dexamethasone; BTZ, bortezomib; L-PAM, melphalan; PSL, prednisone; IXA, ixazomib; ECOG-PS, Eastern Cooperative Oncology Group performance status; ORR, overall response rate; VGPR, very good partial response; PR, partial response; MRD, minimal residual disease; PFS, progression-free survival; DoT, duration of therapy; PD, progressive disease; AEs, adverse events; TEAEs, treatment-emergent AEs; NR, not reached; ND, not described.

## Data Availability

Not applicable.
